# Evolutionary Insights into Taste Perception of the Invasive Pest *Drosophila suzukii*

**DOI:** 10.1534/g3.116.036467

**Published:** 2016-10-19

**Authors:** Cristina M. Crava, Sukanya Ramasamy, Lino Ometto, Gianfranco Anfora, Omar Rota-Stabelli

**Affiliations:** Unit of Agricultural Entomology, Research and Innovation Centre, Fondazione Edmund Mach, 38010 San Michele all’Adige, Trentino, Italy

**Keywords:** gene families, Drosophilidae, gustation, gene birth-and-death, gene duplication, Spotted wing drosophila

## Abstract

Chemosensory perception allows insects to interact with the environment by perceiving odorant or tastant molecules; genes encoding chemoreceptors are the molecular interface between the environment and the insect, and play a central role in mediating its chemosensory behavior. Here, we explore how the evolution of these genes in the emerging pest *Drosophila suzukii* correlates with the peculiar ecology of this species. We annotated approximately 130 genes coding for gustatory receptors (GRs) and divergent ionotropic receptors (dIRs) in *D. suzukii* and in its close relative *D. biarmipes*. We then analyzed the evolution, in terms of size, of each gene family as well of the molecular evolution of the genes in a 14 *Drosophila* species phylogenetic framework. We show that the overall evolution of GRs parallels that of dIRs not only in *D. suzukii*, but also in all other analyzed *Drosophila*. Our results reveal an unprecedented burst of gene family size in the lineage leading to the suzukii subgroup, as well as genomic changes that characterize *D. suzukii*, particularly duplications and strong signs of positive selection in the putative bitter-taste receptor GR59d. Expression studies of duplicate genes in *D. suzukii* support a spatio-temporal subfunctionalization of the duplicate isoforms. Our results suggest that *D. suzukii* is not characterized by gene loss, as observed in other specialist *Drosophila* species, but rather by a dramatic acceleration of gene gains, compatible with a highly generalist feeding behavior. Overall, our analyses provide candidate taste receptors specific for *D. suzukii* that may correlate with its specific behavior, and which may be tested in functional studies to ultimately enhance its control in the field.

Insects rely on stimuli detection to explore the environment for a variety of primary activities, ranging from food localization and mate choice to predator avoidance. Social and environmental chemical cues are perceived and processed through the chemosensory system, resulting in a variety of downstream responses. Thus, genes related to chemosensation are likely to be involved in the evolution of new behaviors and the adaptation to new ecological niches. Taste and smell are the two main mechanisms of chemosensation and they are mostly mediated by three receptor gene families: the highly divergent GR family, found in all arthropods and generally involved in taste; the more recently evolved olfactory receptor (OR) family (Clyne *et al.* 1999; Gao and Chess 1999; Vosshall *et al.* 1999), which is found only in insects and is mainly involved in olfaction (Missbach *et al.* 2014; Robertson 2015); and the ionotropic receptor (IR) family, composed of two distinct subfamilies involved in both taste and olfaction (Benton *et al.* 2009; Croset *et al.* 2010; Koh *et al.* 2014; Stewart *et al.* 2015).

Taste is used by insects for a variety of key processes, such as detection of toxins present in food, identification of secondary metabolites associated with host plants, and recognition of nonvolatile cuticle pheromones. In insects, and likely in various other arthropods, tastant compounds are detected by specialized hair-like structures, called gustatory sensilla, located in different parts of the insect body (*e.g.*, mouthparts, legs, pharynx, and wings) (Stocker 1994). These sensilla house gustatory neurons (GRNs) that express specific transmembrane receptors, which enable the detection of external contact molecules (Freeman and Dahanukar 2015; Joseph and Carlson 2015). Initially discovered in 1999 (Clyne *et al.* 2000), GRs are the first and most extensively studied family of chemoreceptors to be found to be expressed in GRNs in insects. Efforts to elucidate the molecular details of their function have been steadily increasing in the past few years, but to date only a limited number of GRs have been deorphanized in a few insect species (Sato *et al.* 2011; Erdelyan *et al.* 2012; Freeman and Dahanukar 2015; French *et al.* 2015; Joseph and Carlson 2015). In *D. melanogaster*, the GR family includes 60 genes encoding 68 receptor proteins (Robertson *et al.* 2003; Gardiner *et al.* 2008; Almeida *et al.* 2014). Unlike ORs, some GRs are characterized by exhibiting functional plasticity beyond gustatory perception: for example, GR28bD is involved in thermosensation (Ni *et al.* 2013), while GR21a and GR63a are expressed in specific antennal sensilla and are associated with CO_2_ detection (Jones *et al.* 2007; Kwon *et al.* 2007). Such functional diversification is also revealed by the observation that, in *D. melanogaster*, GRs are expressed not only in GRNs, but also in other neurons scattered in different tissues (French *et al.* 2015).

The IRs are a divergent lineage of the ionotropic glutamate receptors (iGluRs), an ancient gene family with a synaptic role in neuronal communication (Benton *et al.* 2009). In *D. melanogaster* there are 57 genes coding for IRs, and they have been divided into two subfamilies depending on their expression, which putatively reflect their ecological role (Croset *et al.* 2010). The first subfamily, antennal IRs (aIRs), is highly conserved among species; it is expressed in olfactory organs and is mainly involved in the detection of air-borne molecules. The second subfamily, dIRs, comprises most the IR genes (around 40 genes in *D. melanogaster*); they are mainly expressed in GRNs and involved with taste, either alone or in association with GRs (Croset *et al.* 2010; Koh *et al.* 2014; Stewart *et al.* 2015). In contrast to aIRs, dIRs evolved under species-specific patterns, with local expansions and/or losses of family members in certain lineages, likely mirroring the natural history of species.

The evolutionary history of GRs and dIRs in *Drosophila* has been studied by exploiting the 12 annotated sequenced genomes (Robertson *et al.* 2003; McBride 2007; McBride *et al.* 2007; Gardiner *et al.* 2008; Croset *et al.* 2010; Almeida *et al.* 2014). In this work, we expanded these studies with the aim of characterizing the evolution of GRs and dIRs in *D. suzukii*, a pest of soft fruits characterized by a peculiar reproductive ecology. This species lays eggs inside ripening unwounded soft fruits, providing an interesting case of shift in oviposition preferences when compared to most other drosophilids, which instead oviposit on fermenting substrates (Rota-Stabelli *et al.* 2013; Asplen *et al.* 2015). We hypothesize that such behavioral change may have been accompanied by duplications and/or losses in taste receptor genes that allowed gravid females to recognize suitable oviposition sites and/or that enabled larvae to feed on fresh fruits. The availability of the genomes of *D. suzukii* and of its closely related species *D. biarmipes*, which does not share oviposition preferences with *D. suzukii*, enabled us to examine the evolutionary diversification of GRs and dIRs associated with this new ecological context. We previously exploited this comparison to reveal a peculiar evolution of ORs and odorant binding proteins (OBPs) in *D. suzukii*, and to identify various genes and ligands that likely play an active role in its attraction toward fresh rather than fermenting fruits (Ramasamy *et al.* 2016). The role of taste receptors in *D. suzukii* behavior is, on the other hand, still completely unexplored. Here, we have characterized GR and dIR families in *D. suzukii* and *D. biarmipes*, studied their evolution in a 14 *Drosophila* phylogenetic framework, and further analyzed expression profiles of duplicate genes in *D. suzukii*. We used our results to address three major questions. Is there any evidence of lineage-specific differentiation of GR or dIR repertoires in *D. suzukii* that may correlate with its peculiar behavior? Are the two taste perception modalities (GRs *vs.* dIRs) evolving with similar patterns? Finally, do recently duplicated genes have nonredundant spatio-temporal expression patterns that avoid a possible overlap in their function? Our results revealed an unusual burst of gene copies in both GR and dIR families in the branch leading to the suzukii subgroup (which includes both *D. suzukii* and *D. biarmipes*), whereas only few genomic changes uniquely characterize *D. suzukii* taste gene repertoire. We found evidence of a similar evolution pattern for GRs and dIRs not only in *D. suzukii*, but also in all the analyzed *Drosophila*. Lastly, reverse transcription PCR (RT-PCR) showed a tissue-specific expression pattern for some duplicate gene families which supports a role of some of them as pheromone receptors. Overall, our dataset is the first describing the complete *D. suzukii* taste receptor repertoire and suggests that the diversification of bitter receptors has played a central role in the evolution of the suzukii subgroup lineage.

## Materials and Methods

### Annotation of GRs and IRs

We used tBLASTn (cut-off values 10^−5^) to iteratively search against *D. suzukii* (Chiu *et al.* 2013; Ometto *et al.* 2013) and *D. biarmipes* genomes using *D. melanogaster* GRs and dIRs (obtained from FlyBase release FB2015_02) as query sequences. Coding sequences (CDS) were manually predicted *in silico* by mapping exons identified in tBLASTn searches using BioEdit (Hall 1999). Introns (following the GT-AG rule) were removed and the remaining sequences were checked for an in-frame coding sequence. *D. suzukii* or *D. biarmipes* sequences with indels leading to a premature stop codon were considered pseudogenes. In *D. suzukii*, GRs and IRs were retrieved from both the Italian (Ometto *et al.* 2013) and the American (Chiu *et al.* 2013) genomes. Paralogous duplications were cross-checked between the two genomes; genes that were represented by more copies located in different scaffolds and in only one genome, and which diverged for only a few SNPs, were considered allelic variants that were not well assembled during genome assembly. Genes were named based on the reconstructed phylogenetic tree (see below) and following the *D. melanogaster* nomenclature, while adding a two-letter prefix corresponding to the species’ names. Orthologs in *D. suzukii* and *D. biarmipes* were named with consecutive numbers: for example, *IR47a* has two copies in *D. suzukii* and *D. biarmipes*, which were named as *DsIR47a1*, *DsIR47a2*, *DbIR47a1*, and *DbIR47a2*. Paralogs whose orthologs could not be clearly identified in the other species were named with consecutive numbers after a point: for example, *GR59d8* has three copies in *D. suzukii* which were named *DsGR59d8.1*, *DsGR59d8.2*, and *DsGR59d8.3*. We did not rename genes with previously published names (*e.g.*, in *D. ananassae IR94j* or *IR94k*).

### Phylogenetic analysis

Nucleotide sequences of GRs from *D. melanogaster*, *D. pseudoobscura*, *D. ananassae*, and *D. erecta* were downloaded from FlyBase (release FB2015_02) according to datasets used before (Gardiner *et al.* 2008; Almeida *et al.* 2014), whereas dIR sequences were obtained from Croset *et al.* (2010) (Supplemental Material, Table S2). These species were chosen to recreate the taxon sampling of Ramasamy *et al.* (2016), which proved to be useful for comparative studies. In cases of mis-annotated genes in species other than *D. suzukii* and *D. biarmipes*, sequences retrieved from databases were manually reannotated from whole-genome sequencing data to unify gene structure prediction across species (Table S2). These sequences, together with *D. suzukii* and *D. biarmipes* genes, were aligned with TranslatorX (Abascal *et al.* 2010) using the Muscle algorithm (Edgar 2004), and the resulting alignments were manually checked and edited. Maximum likelihood amino acid-based trees were then calculated with RAxML (Stamatakis 2014) using the PROTGAMMA+LG+F model and bootstrapping the dataset with 100 pseudoreplicates. Trees were viewed and graphically edited using iTOL (Letunic and Bork 2007). Pseudogenes were excluded from the alignments.

### Gene birth and death estimation

To infer the number of GRs and dIRs duplications and losses along *Drosophila* phylogeny, we used Badirate (Librado *et al.* 2012), which reconciles gene trees onto the species tree. The species tree, which included divergence dates for 14 drosophilid species, was the one proposed by Ometto *et al.* (2013). The data matrix with the gene numbers for each species was taken and slightly modified from Almeida *et al.* (2014), except for data for *D. persimilis* that were added from Croset *et al.* (2010) and Gardiner *et al.* (2008) (Table S3). To obtain β (birth rate) and γ (death rate) estimates we applied the BDI-FR-CML method, which uses a full maximum-likelihood approach and assumes independent evolution for each branch along each lineage.

### Analysis of selective forces

We used PAML 4.7 (Yang 2007) to infer the rate of nonsynonymous, *d_N_*, and synonymous nucleotide substitution, *d_S_*, as well as the level of selective pressure acting on a gene (ω *= d_N_*/*d_S_*). We first created multiple sequence alignments of orthologous genes from six *Drosophila* species (*D. suzukii*, *D. biarmipes*, *D. melanogaster*, *D. erecta*, *D. pseudoobscura*, and *D. ananassae*) with PRANK (Löytynoja and Goldman 2005) and TranslatorX. In case of paralogs in species other than *D. suzukii* or *D. biarmipes*, we used the most conserved isoform compared to the other lineages. In case of duplications in *D. suzukii* and *D. biarmipes*, the analysis was repeated for each of the paralogs using the closest ortholog from the other species. Pseudogenes were excluded from the analysis. To estimate ω values in *D. suzukii* and *D. biarmipes*, PAML was run using the “free-ratio” model, which allows branch-specific values for ω over all branches of the unrooted phylogenetic tree. Tree topology was taken from Ometto *et al.* (2013). To evaluate heterogeneity in the selective pressure on *D. suzukii* or *D. biarmipes*, we used a branch test that compared the likelihood of a model that assumed a single ω across branches (model = 0 and NSsites = 0) to a second that assumed a distinct ω for the focal branch (*D. suzukii* or *D. biarmipes*). To identify the occurrence of positive selected sites along *D. suzukii* or *D. biarmipes* branches, we used the branch-site test (branch-site model A, test 2; model = 2 and NSsites = 2; null model has parameters fix_ ω = 1, ω = 1; the positive selection model fix_ ω = 0, ω = 1). In both branch and branch-site tests, the value of twice the difference between the two alternative likelihoods was tested using a χ^2^ test with one degree of freedom. To account for multiple testing, we estimated the false discovery rate (FDR) using the Benjamini and Hochberg correction (Benjamini and Hochberg 1995).

### RT-PCR

Gene expression analysis was carried on *D. suzukii* adults from a population collected in Trento Province (Italy) and maintained in our laboratory on a standard *Drosophila* semiartificial diet (*Drosophila* species stock center, https://stockcenter.ucsd.edu/info/food_cornmeal.php) at a temperature of 23–25°, relative humidity of 65 ± 5%, and 16L:8D photoperiod. We dissected flies from both sexes using forceps, and we pooled males and females to obtain four different samples: heads (*n* = 20), thoraxes (including wings and legs; *n* = 10), abdomens (*n* = 10), and antennae (*n* = 300 pairs). A fifth sample was composed of third instar larvae (*n* = 10) that were processed as whole body samples. Additionally, two more samples consisting of female forelegs and male forelegs (*n* = 100 legs each) were prepared. All samples were placed immediately in cold RNAlater (LifeSciences) and stored at −80° until crushed in Trizol (LifeSciences) using Tissue Lyser II (QIAGEN). Total RNA was extracted followed the Trizol manufacturer’s instructions. Extracted RNA was treated with RNAse-free DNAse (LifeSciences) and then used for first strand cDNA synthesis using Superscript RT III (LifeSciences). One µl of cDNA diluted 1:5 was amplified by PCR with GreenTaq (Promega) according to the manufacturer’s instructions using 32 amplification cycles. To control for genomic DNA contamination, each batch of total RNA underwent a parallel mock reverse transcription step in which the reverse transcriptase was omitted. The cDNA quality was checked by tubulin amplification. PCR primers (listed in Table S4) were first checked against genomic DNA. Two biological replicates were done for each sample, and each amplification was repeated at least twice.

### Data availability

**Figure 1:**
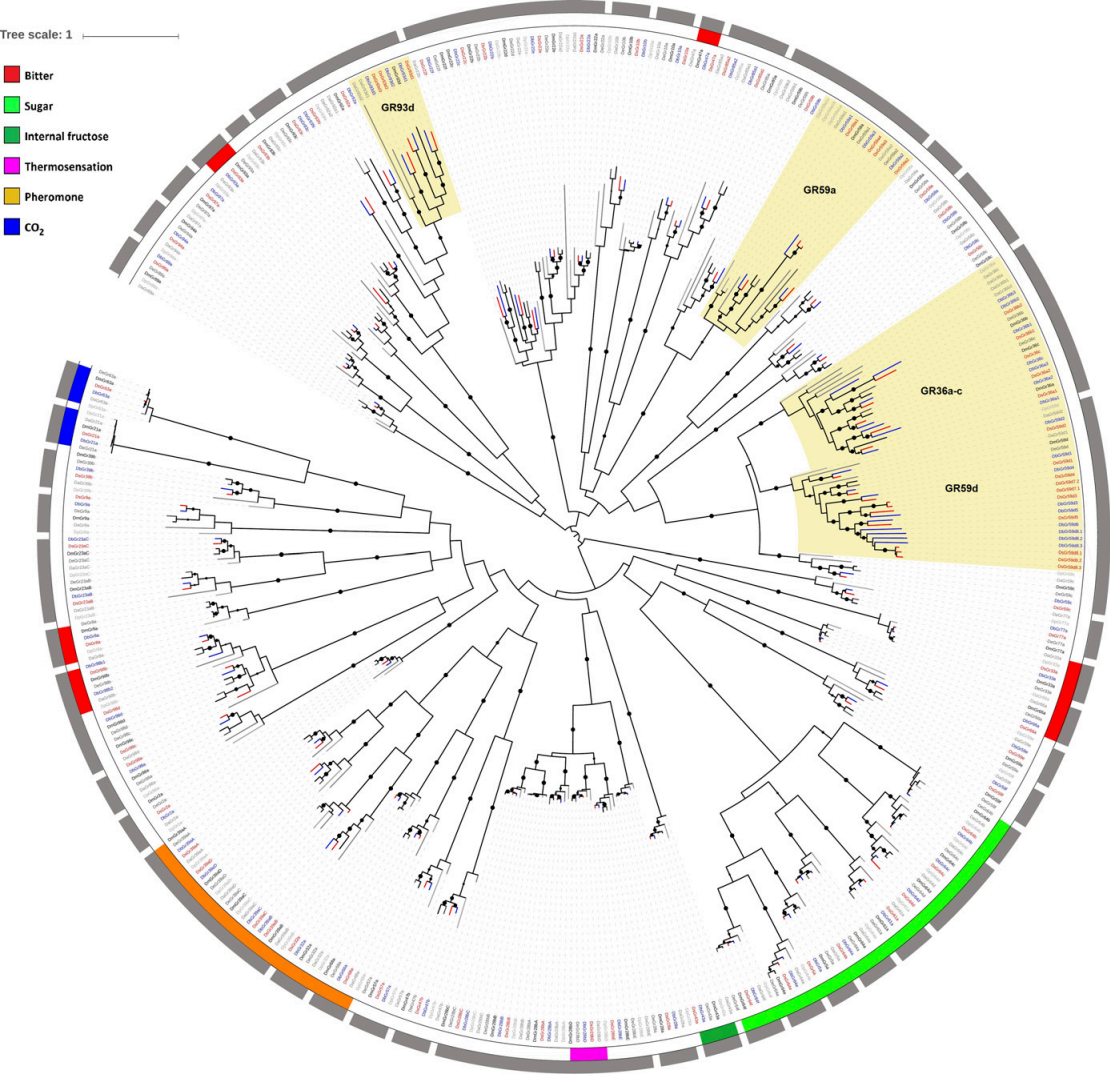
Gene phylogeny of GRs from six drosophilid genomes. Maximum likelihood tree calculated with RAxML and based on Muscle nucleotide alignment implemented in TranslatorX. Bootstrap support is out of 100 replicates and support > 80 is indicated by black dots whose sizes are according to bootstrap values. Gray half circles identify the gene groups used in birth-death analysis. Branches highlighted in red show *D. suzukii* genes, and branches highlighted in blue show *D. biarmipes* genes. *D. melanogaster* genes are colored in black whereas other *Drosophila* lineages are shown with different gray shades. Color legends refer to GRs whose function has been deorphanized in *D. melanogaster* (Joseph and Carlson 2015). Clades highlighted in yellow have undergone specific gene expansion in the suzukii subgroup. GRs, gustatory receptors.

**Figure 2:**
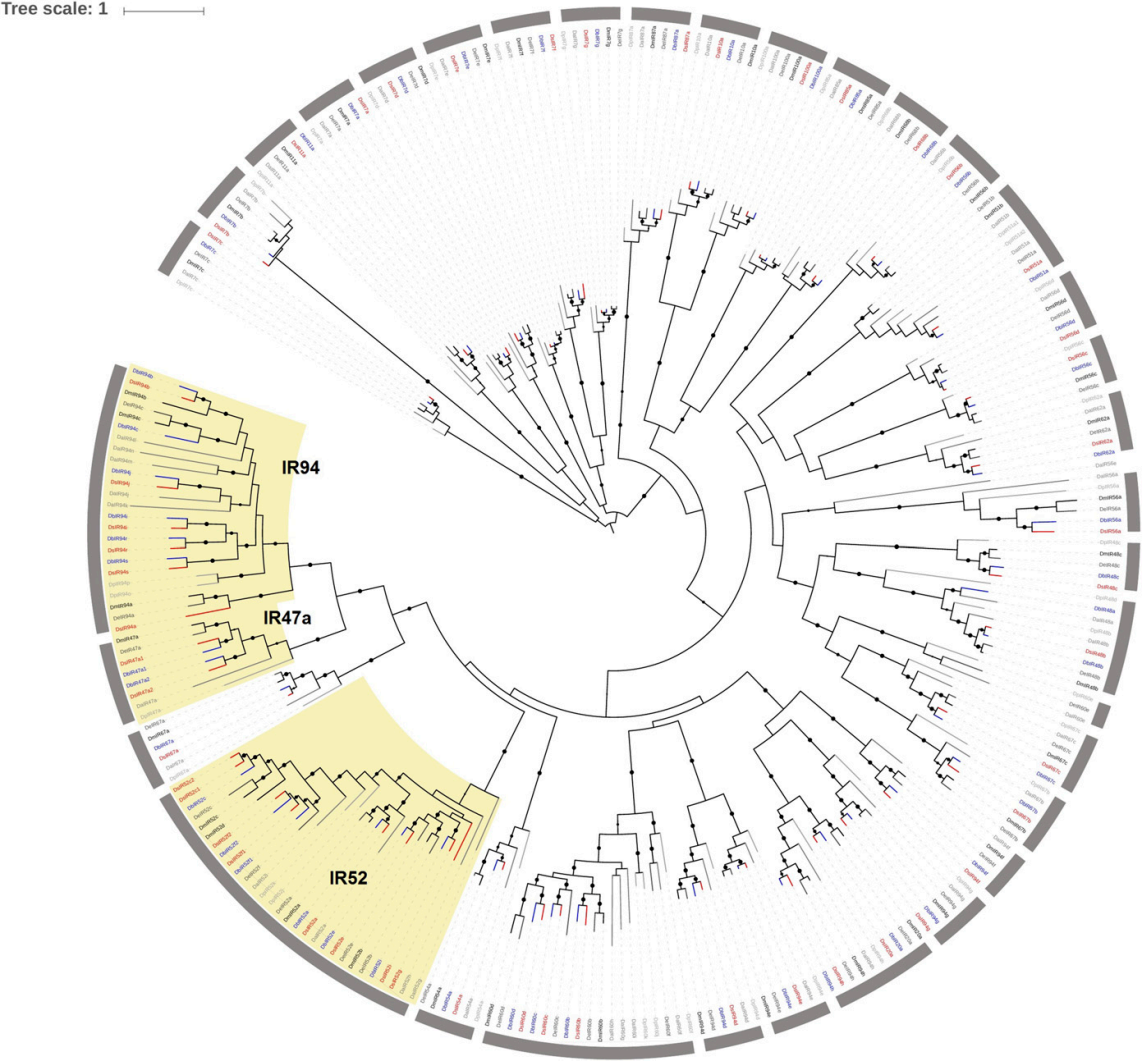
Gene phylogeny of dIRs from six drosophilid genomes. Maximum likelihood tree calculated with RAxML and based on Muscle nucleotide alignment implemented in TranslatorX. Bootstrap support is out of 100 replicates and support > 80 is indicated by black dots whose sizes are according to bootstrap values. Gray half circles identify the gene groups used in birth-death analysis. Branches highlighted in red show *D. suzukii* genes, and branches highlighted in blue show *D. biarmipes* genes. *D. melanogaster* genes are colored in black whereas other *Drosophila* lineages are shown with different gray shades. Clades highlighted in yellow have undergone gene expansion in suzukii subgroup. dIRs, divergent ionotropic receptors.

Sequence data are presented as File S1 and File S2, and their scaffold locations are listed in Table S1. Alignments used to build Figure 1 and Figure 2 are contained in File S3 and File S4. File S5 contains supplementary figures.

## Results

### Annotation of GRs and dIRs in the genomes of D. suzukii and D. biarmipes

We manually annotated 77 GRs in *D. suzukii* and 76 in *D. biarmipes*. Genomic evidence of alternative splicing was found for three genes in both species (*GR23a*, *GR28b*, and *GR39a*), bringing the total of predicted GR proteins to 85 (File S1) and 84 (File S2), respectively; in *D. biarmipes*, we identified three pseudogenes (Table S1). All genes have identical intron–exon structures in the two species: exons range from 1 to 9, but most genes have one or two exons, and only two genes are intronless (*GR94a* and *GR68a*) (Table S1).

We identified 50 dIRs in the *D. suzukii* genome and 49 in the *D. biarmipes* one, and a pseudogene in *D. biarmipes* (File S1, File S2, and Table S1). Similar to GRs, intron–exon structures of dIRs were the same for both species. dIRs were characterized by less introns than GRs; most genes (around 70%) were intronless, and the remaining ones had one to four introns per gene (Table S1).

### GR and dIR evolution in the suzukii subgroup

To obtain a detailed insight into gene duplication and loss in *D. suzukii*, we inferred the phylogenetic relationships of GRs and dIRs from *D. suzukii* to that of five other *Drosophila* species (*D. biarmipes*, *D. melanogaster*, *D. erecta*, *D. pseudoobscura*, and *D. ananassae*) (Figure 1 and Figure 2). Using the *D. melanogaster* orthologs as reference, 66% of GRs have a one-to-one orthologous relationship across the six species, while copy number variation occurs for the remaining genes. In particular, 15 genes are missing in at least one of the six species, whereas 10 have multiple copies in at least one species. Among dIRs, most copy number variation events are mostly species-specific (*i.e.*, families IR52, IR60, and IR94). Considering only the 41 dIRs present in *D. melanogaster* genome, 68% of them have a one-to-one orthologous relationship across the six species studied.

**Figure 3:**
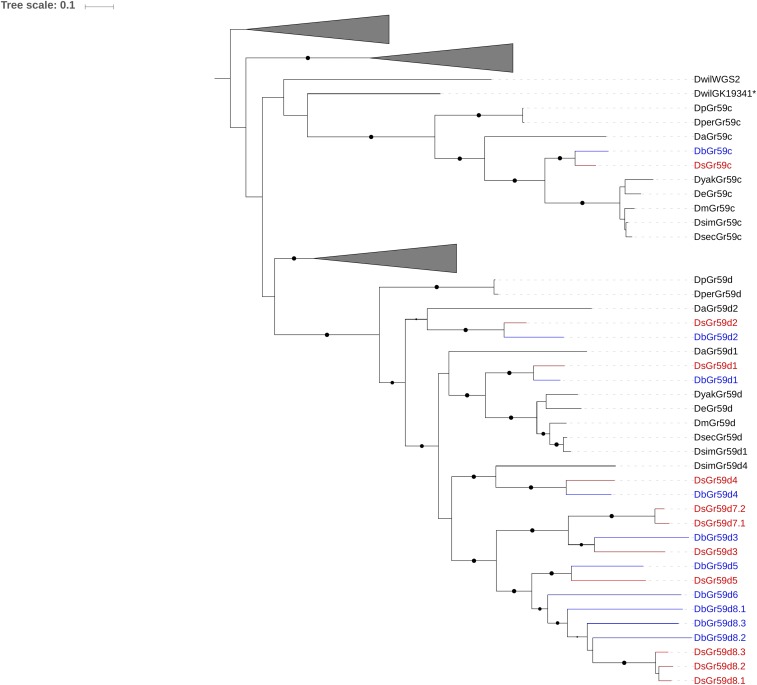
Gene phylogeny of GR59c and GR59d families from 14 *Drosophila* species. Maximum likelihood tree calculated with RAxML and based on Muscle nucleotide alignment implemented in TranslatorX. Phylogenetic relationships of all genes included in gene family GR59c-d used in birth-death analysis are displayed in the tree. Bootstrap support is out of 100 replicates and support > 80 is indicated by black dots whose sizes are according to bootstrap values. Branches highlighted in red show *D. suzukii* genes, and branches highlighted in blue show *D. biarmipes* genes. Branches without *D. suzukii* or *D. biarmipes* genes are collapsed. GRs, gustatory receptors.

Five GR genes (*GR36a*, *GR36b*, *GR59a*, *GR59d*, and *GR93d*) have multiple copies in *D. suzukii* and *D. biarmipes* genomes compared to other drosophilids (a total of 17 and 16 more genes than *D. melanogaster*, respectively) (Figure 1 and Table 1). Based on their orthological relationships, we observed that some triplications and duplications (*GR36a*, *GR36b*, *GR59a*, and *GR93d*) are clearly shared between *D. suzukii* and *D. biarmipes*, and thus they likely originated in the common ancestor of the suzukii subgroup. Other duplications or losses are instead species-specific, such as that observed for *GR59a4*, which is present only in *D. suzukii*. The duplication pattern of *GR59d* is particularly complex (Figure 3). Some duplicated copies of *GR59d* show a one-to-one orthologous relationship, and their synteny is conserved between the two species (*GR59d2*, *GR59d3*, *GR59d4*, and *GR59d5*) (Figure S1) indicating

**Table 1:** Representative GR and dIR genes characterized by duplication events and signatures of differential selective pressure in *D. suzukii*

Gene	Type of Duplication	Significant Branch Test at FDR < 0.05	Expression in *D. suzukii*	Ortholog Expression in *D. melanogaster*
*GR36a2*	Suzukii subgroup	ω = 1.25, FDR < 0.0001	Head	*GR36a*: labellar sensilla*^a^*
*GR36b2*	Suzukii subgroup	ω = 1.02, FDR = 0.021	Head	*GR36b*: labellar sensilla*^a^*, larval sensilla innervating terminal distal group*^b^*
*GR59a3*	Suzukii subgroup	ω = 0.23, FDR = 0.030	ND	*GR59a*: labellar sensilla*^a^*, foreleg sensilla*^c^*, larval sensilla innervating terminal distal group*^b^*
*GR59a4*	Unique to *D. suzukii*	ω = 0.27, FDR = 0.021	ND	
*GR59d2*	Suzukii subgroup	ω = 0.48, FDR = 0.011	Head, foreleg, larva	*GR59d*: labellar sensilla*^a^*, foreleg sensilla*^c^*, larval sensilla innervating terminal distal group*^b^*
*GR59d3*	Suzukii subgroup	ω = 1.2, FDR < 0.0001	Head, abdomen, larva	
*GR59d5*	Suzukii subgroup	ω = 0.66, FDR < 0.0001	Head	
*GR59d7.1*	Unique to *D. suzukii*	ω = 0.41, FDR = 0.0066	ND	
*GR59d7.2*	Unique to *D. suzukii*	ω = 0.39, FDR = 0.0114	ND	
*GR59d8.1*	Unique to *D. suzukii*	ω = 1.00, FDR < 0.0001	Head, larva	
*GR59d8.2*	Unique to *D. suzukii*	ω = 1.72, FDR < 0.0001	Head, larva	
*GR59d8.3*	Unique to *D. suzukii*	ω = 1.28, FDR < 0.0001	Head, larva	
*IR47a2*	Suzukii subgroup	ω = 0.57, FDR = 0.0105	Abdomen, thorax, foreleg, head, larva	*IR47a*: labellar sensilla*^d^*, foreleg sensilla*^d^*

FDR, false discovery rate; ND not detected.

a Weiss *et al.* 2011.

b Ling *et al.* 2014.

c Kwon *et al.* 2011.

d Koh *et al.* 2014.

that duplication events occurred in the common ancestor of suzukii subgroup. Within the same gene cluster, *D. biarmipes* possesses an additional copy (*GR59d6*) (Figure S1) that is not present in the *D. suzukii* genome. Another copy of *GR59d*, *GR59d7*, also originated from duplication in the common ancestor of the suzukii subgroup and later diverged in the two species: in *D. biarmipes GR59d7* is likely a pseudogene, whereas in the *D. suzukii* genome it is present in two paralogous (functional) copies. *GR59d8* evolution is extremely problematic; there are three distinct copies in *D. suzukii* and in *D. biarmipes* (plus a pseudogene in the latter). In both species, the three copies cluster together in a different scaffold to those containing *GR59d1–6* and *GR59d7* (Figure S1). Although not well resolved, their phylogenetic affinities inferred by ML tree (Figure 3) suggest various rounds of duplication in the common ancestor of *D. suzukii* and *D. biarmipes* followed by various rounds of deletion in *D. suzukii*, and a recent triplication in *D. suzukii*. Alternatively, it is possible that the three copies in *D. suzukii* are evolving in a concerted fashion and experienced gene conversion by homologous recombination, therefore showing high similarity among them.

Three dIR families (*IR47a*, *IR52*, and *IR94*) belonging to the IR20a clade (Koh *et al.* 2014) experienced higher turnover in either *D. suzukii* and *D. biarmipes* genomes compared with the rest of *Drosophila* species (Figure 2); IR47a2 and IR52f2 are two new duplicates in both species whereas IR52i is a receptor found only in *D. suzukii* and *D. biarmipes* (Figure S2). *IR52c* is duplicated only in *D. suzukii*, while *D. biarmipes* does not have *IR52g* (Figure 2 and Figure S2). Finally, the IR94 family experienced multiple cases of gene loss and duplication; while *D. biarmipes* and *D. suzukii* possess multiple orthologs copies compared to the other *Drosophila* species (*IR94i*, *IR94r*, and *IR94s*), *D. suzukii* specifically lost *IR94c* whereas *D. biarmipes* lost *IR94a* (Figure 2 and Figure S3).

### Outburst of taste receptor duplication in the branch leading to the suzukii subgroup

**Figure 4:**
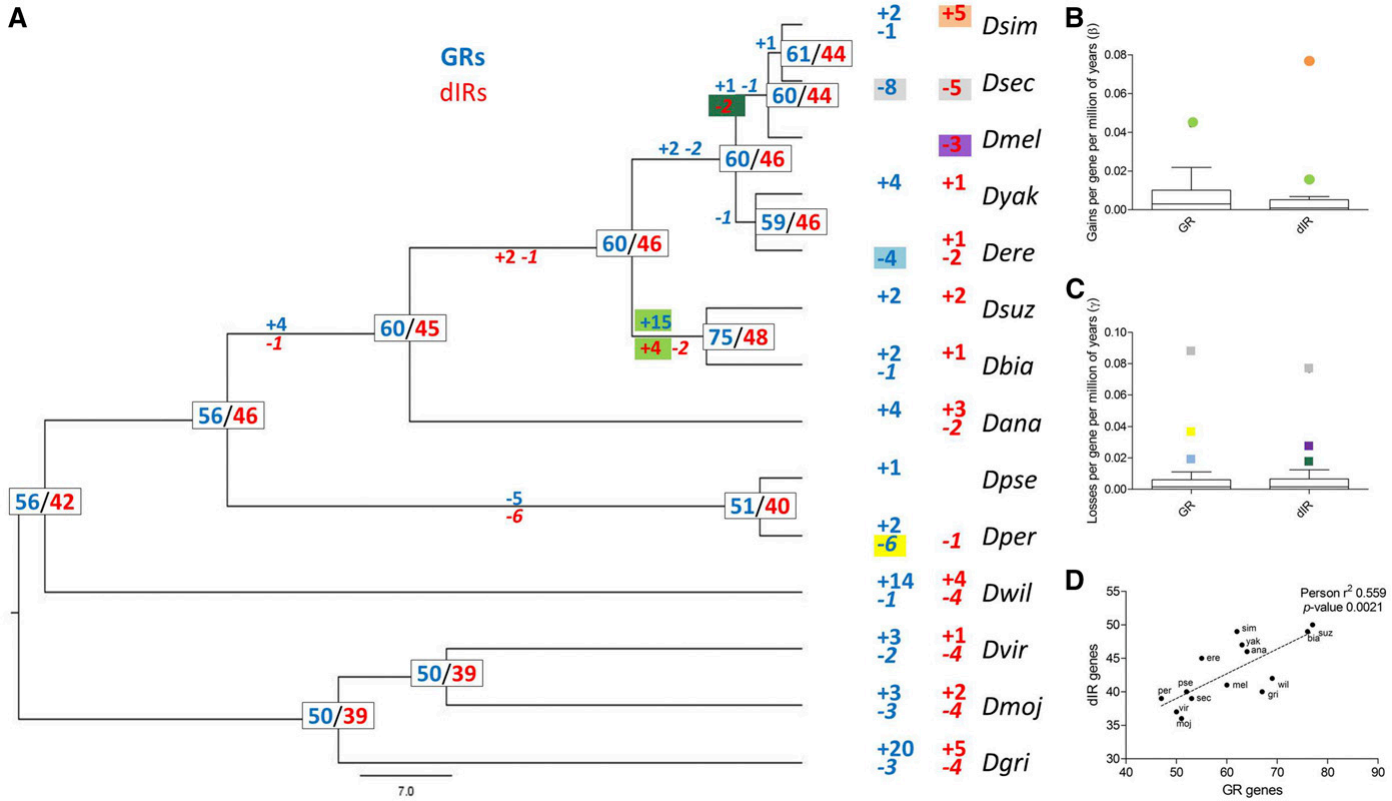
Gene loss and gain in GR and dIR drosophilid repertoires. (A) Estimates of gene birth and death on internal nodes of *Drosophila* phylogeny [based on Ometto *et al.* (2013)]. Numbers on each node represent the estimated number of genes at that internal node whereas numbers on branches represent gene gains and losses. Gains and losses referred to birth rates (β) or death rates (γ), which are an outlier in Tukey boxplots, are highlighted with colors referring to the corresponding Tukey boxplot. (B) Tukey boxplot showing birth rates (β) calculated by Badirate under the BDI-FR-CML model. (C) Tukey boxplot showing death rates (γ) calculated by Badirate under the BDI-FR-CML model. (D) Correlation among number of GR and dIR gene copies along *Drosophila* phylogeny. dIR, divergent ionotropic receptor; GR, gustatory receptor.

To quantify the number of GR and dIR gain and loss events, we analyzed the gene phylogeny in the context of species phylogeny using Badirate (Figure 4). Because we could not resolve the orthological relationships of *GR59d7* duplicates with confidence, we have used a conservative view that implies duplications in the common ancestor of *D. suzukii* and *D. biarmipes*. Badirate estimated that along the whole *Drosophila* phylogeny, GRs experienced 38 losses (global death rate of 0.0022 losses per gene per millions of years, l/g/m) and 87 gains (global birth rate of 0.0045 gains per gene per millions of years, g/g/m), whereas dIRs experienced 43 losses (global death rate of 0.0029 l/g/m) and 38 gains (global birth rate of 0.0025 g/g/m) (Figure 4A). Notably, the highest birth rate for GRs occurs in the branch leading to the suzukii subgroup, where we can observe the gain of 15 GRs (birth rate 0.0439 g/g/m, tenfold higher than the average, Figure 4B). On the same branch, we can observe the second highest birth rate for dIRs (the highest is for *D. simulans*), characterized by four dIR gains (birth rate 0.0153 g/g/m, sixfold higher than the average, Figure 4B). Particularly high death rates occur for both gene families in the branch leading to the specialist *D. sechellia* (Figure 4C).

Interestingly, in the 14 drosophilid genomes, the family size of GRs correlates with the family size of dIRs (Figure 4D). *D. suzukii* and *D. biarmipes* are the species with the highest number of genes in both families, whereas the two species from the obscura group (*D. pseudoobscura* and *D. persimilis*), the specialists *D. sechellia* and *D. mojavensis*, and *D. virilis* have the lowest number of both GRs and dIRs.

### Signatures of different selective pressure on genes encoding taste receptors in D. suzukii and D. biarmipes

**Figure 5:**
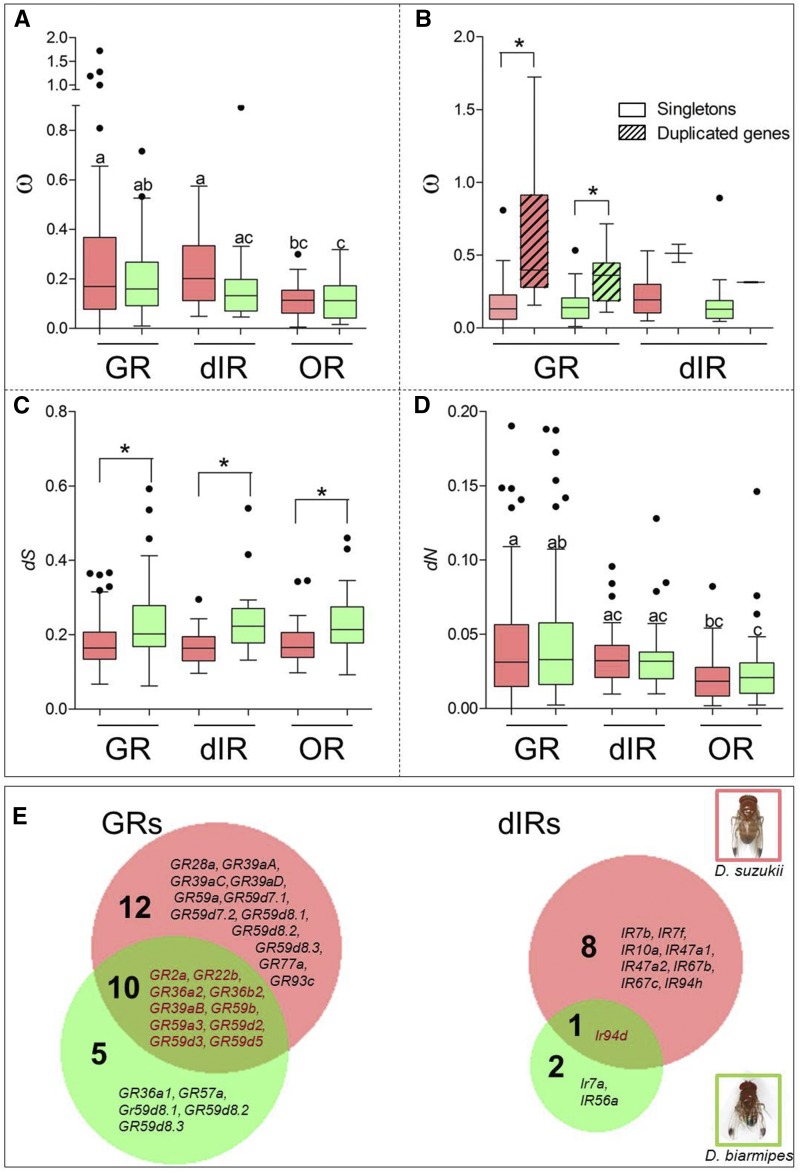
Selective pressure acting on *D. suzukii* and *D. biarmipes* chemoreceptor genes. All boxplots were built using values estimated for genes present in the six lineages. OR dataset was taken from Ramasamy *et al.* (2016). (A) Tukey boxplots showing *d_N_/d_S_* (ω) for GRs, dIRs, and ORs in *D. suzukii* (pink) and *D. biarmipes* (green). Significant differences among groups were highlighted by Kruskal–Wallis test (*H* = 25.33, 5 d.f., and *P* < 0.0001) followed by Dunn’s multiple comparison *post hoc* test (different letters indicate significance levels at *P* < 0.05). (B) Tukey boxplots showing the *d_N_/d_S_* ratios (ω) for singleton (empty plots) or duplicate (striped plots) GRs and dIRs in *D. suzukii* (pink) and *D. biarmipes* (green). Differences between singleton and duplicate gene were tested only for GRs in both *D. suzukii* and *D. biarmipes* by Wilcoxon test using a Bonferroni-corrected *P*-value (*P* < 0.025). (C) Tukey boxplots showing synonymous substitution rate (*d_S_*) for GRs, dIRs, and ORs in *D. suzukii* (pink) and *D. biarmipes* (green). Significant differences among groups were highlighted by Kruskal–Wallis test (*H* = 39.61, 5 d.f., and *P* < 0.0001) followed by Dunn’s multiple comparison *post hoc* test (*P* < 0.05). (D) Tukey boxplots showing nonsynonymous substitution rate (*d_N_*) for GRs, dIRs, and ORs in *D. suzukii* (pink) and *D. biarmipes* (green). Significant differences among groups were highlighted by Kruskal–Wallis test (*H* = 23.89, 5 d.f., and *P* < 0.0005) followed by Dunn’s multiple comparison *post hoc* test (different letters indicate significance levels at *P* < 0.05). (E) Venn diagrams depicting the number of genes under a differential selective pressure in *D. suzukii* (pink) and *D. biarmipes* (green) identified by PAML branch-test at FDR < 0.05. dIR, divergent ionotropic receptor; FDR, false discovery rate; GR, gustatory receptor; OR, olfactory receptor.

We studied the overall selective pressure acting on GRs and dIRs in *D. suzukii* and *D. biarmipes* by examining the *d_N_/d_S_* ratio (ω) at each locus. All tested GRs and dIRs in both species are under a gene-wide moderate to strong purifying selection regime (ω < 1), with the notable exception of six duplicate genes in *D. suzukii* (from the GR59d, GR36a, and GR36b families) and one (GR36b2) in *D. biarmipes* (Figure 5A, Table 1, and Table S5). In both species, ω ratios are always higher for duplicate genes compared to nonduplicate ones (Figure 5B). Within both species, the level of selective pressure of GRs is similar to that of dIRs (medians of 0.170 and 0.202 for *Ds*GRs and *Ds*dIRs, respectively; 0.159 and 0.132 for *Db*GR and *Db*dIR, respectively), and both are larger than those estimated for ORs (medians of 0.114 and 0.112 for *Ds*OR and *Db*OR, respectively). Within each class of receptors, no differences between species are evident (Figure 5A). The synonymous substitution rate (*d_S_*) does not vary among GRs, dIRs, and ORs within each species (medians for *D. suzukii*: *Ds*GR = 0.164, *Ds*dIR = 0.164, and *Ds*OR = 0.166; medians for *D. biarmipes*: *Db*GR = 0.202, *Db*dIR = 0.223, and *Db*OR = 0.214), whereas *d_S_* values in *D. biarmipes* are higher than *D. suzukii* for all receptors (Figure 5C). The nonsynonymous substitution rate (*d_N_*) does not vary among species and taste receptors (medians: *Ds*GR = 0.031, *Ds*dIR = 0.032, *Db*GR = 0.033, and *Db*dIR median = 0.032), whereas ORs have the lowest values in both species (medians: *Ds*OR = 0.018 and *Db*OR = 0.021) (Figure 5D).

Branch tests identified 28 GRs in *D. suzukii* (22 at FDR < 0.05) and 23 in *D. biarmipes* (15 at FDR < 0.05) that are evolving under differential selective pressure in either of these species compared to the rest of the phylogeny. Ten of these genes are shared between the two species, seven of which are duplicate genes (Figure 5E and Table S5). Among dIRs, 12 genes in *D. suzukii* (nine at FDR < 0.05) and five in *D. biarmipes* (three at FDR < 0.05) have signatures of differential selection pressure, and one of them is shared between the two species (Figure 5E). This corresponds to 38 and 29% of GRs and dIRs under differential selection pressure, respectively, suggesting a very dynamic selective regime in this class of genes. To test if few sites inside GRs or dIRs are evolving under positive selection (but are masked by purifying or relaxed selection acting on the other parts of the gene), we applied a branch-site test and obtained evidence for site-specific selection in *D. suzukii* and *D. biarmipes*. Eight GRs (one at FDR < 0.05) and two dIRs (none at FDR < 0.05) were detected as having sites under positive selection in *D. suzukii*, whereas in *D. biarmipes* five GRs (two at FDR < 0.05) and one dIR (at FDR < 0.05) were identified (Table S5).

### Spatio-temporal expression of duplicate GRs and IRs in D. suzukii

**Figure 6:**
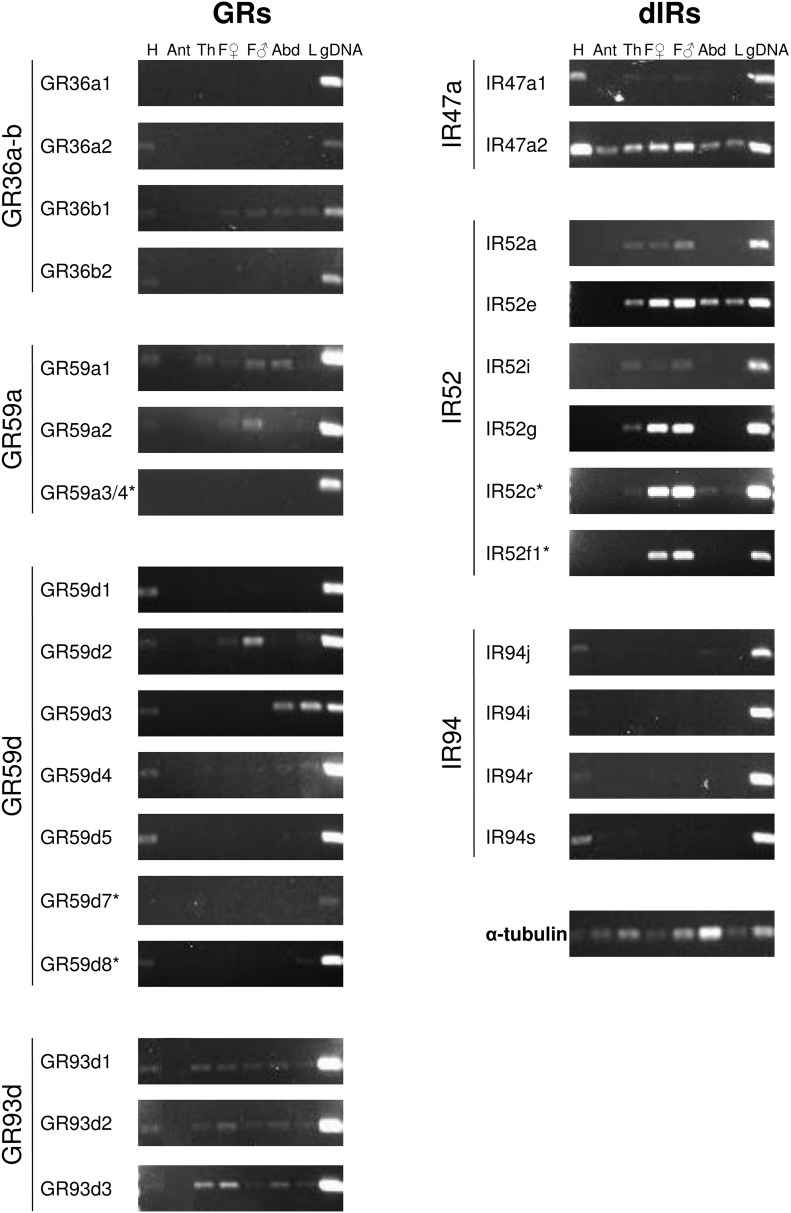
Spatial and temporal expression of duplicate GRs and dIRs in *D. suzukii*. Gene expression analysis in different *D. suzukii* adult tissues and in third instar larvae was carried out by RT-PCR. The experiment was performed twice with at least two technical replicates per sample. Samples were run on 1% agarose gel stained with ethidium bromide. Asterisks indicate primer pairs that were not able to discriminate between isoforms. Gel bands for *GR93d1*, *GR93d2*, and *GR93d3* are shown in a different order than gel loading. Abd, abdomen; Ant, antennae; dIR, divergent ionotropic receptor; F, forelegs; gDNA, genomic DNA; GR, gustatory receptor; H, head; L, larvae; RT-PCR, reverse transcription polymerase chain reaction; Th, thorax.

Expression patterns of the genes that underwent duplication in *D. suzukii* are reported in Figure 6. Family IR52 is mainly expressed in the thorax, specifically in forelegs of both females and males. One IR52 (*DsIR52e*) is also clearly expressed in larvae; interestingly, no members of this family are expressed in adult heads. *DsIR47a2* shows the broadest expression pattern since it is expressed in all tissues and stages tested, whereas expression of *DsIR47a1* was mainly detected in the head (a slight band is also observed for thorax and forelegs). Families IR94, GR36a, GR36b, GR59a, and GR59d are mainly expressed in heads, although some specific members are expressed in other tissues or during the larval stage; the three members of the GR59d family (GR59d8.1, GR59d8.2, and GR59d8.3) under positive selection are expressed in the head and in larvae (Table 1). Finally, all the three members of family GR93d are ubiquitously expressed in heads (but not antennae), thorax, and abdomen, as well as in larvae. None of the tested dIRs or GRs (except for IR47a2) were detected in antennae.

## Discussion

### Evolution of taste genes on the Drosophila phylogeny

In the last few years, the availability of numerous *Drosophila* genomes has allowed several comparative studies to be carried out that have described the evolution of chemosensory genes along the *Drosophila* phylogeny. Their results have highlighted lineage-specific expansions and contractions that have occasionally been associated with phenotypical differences between species (Guo and Kim 2007; McBride 2007; McBride *et al.* 2007; Gardiner *et al.* 2008; Croset *et al.* 2010; Almeida *et al.* 2014; Ramasamy *et al.* 2016). Some of these studies suggested that the evolutionary properties of genes encoding chemosensory receptors correlate with their putative function. For example, genes involved in taste, but belonging to phylogenetically distinct families (GRs and dIRs), evolve under evolutionary constraints that are different from those of genes involved with smell (such as ORs and aIRs) (Croset *et al.* 2010). Our results confirm this trend by further showing that GRs and dIRs are characterized by a similar lineage-specific turnover rate along the *Drosophila* phylogeny (Figure 4). Other confirmation comes from our molecular evolution studies; in *D. suzukii*, GRs and IRs are characterized by a more relaxed selective pressure than that observed in ORs (Figure 5A).

Overall, our average ω values are consistent with purifying selection acting in both chemosensory gene families, as reported for other *Drosophila* (Gardiner *et al.* 2008; Croset *et al.* 2010). Furthermore, faster molecular evolution was found for genes that underwent gene duplication, supporting the hypothesis that recently duplicate genes experience lower selective constraints and thus can be a source of genetic novelty (Lynch and Conery 2000; Kondrashov *et al.* 2002).

### Outburst of taste receptors supports a generalist feeding behavior in the suzukii subgroup

Previous work in *Drosophila* and other insect species indicated that GR losses are often associated with host specialization. The specialists *D. sechellia*, which feeds only on Morinda fruits, and to a lesser extent *D. erecta*, which feeds on *Pandanus* spp., are losing GRs more rapidly than generalists (McBride 2007; McBride *et al.* 2007). In the butterflies of the genus *Heliconius*, whose members are specialized on different *Passiflora* species, there have been a number of species-specific gene losses in bitter-related GRs (Briscoe *et al.* 2013). In all such cases, it seems that specialization on a novel host plant is generally associated with a contraction of the GR family. *D. suzukii*, although being characterized by a peculiar larval feeding ecology, cannot be defined as a specialist; adults feed on fermenting substrates like other *Drosophila* generalists, and females lay eggs in a great variety of fruits. Accordingly, *D. suzukii* is not characterized by a reduced number of taste receptors as is seen in specialist species, but rather by the highest number of both GRs and IRs among the sampled *Drosophila* lineages. This is compatible with a generalist feeding habit, as expanded molecular taste machinery would allow perception of a large assortment of tastants from a wide variety of food sources. This is supported by experimental evidence showing that *D. suzukii* can oviposit in extremely different fruit species (Lee *et al.* 2011; Yu *et al.* 2013; Poyet *et al.* 2015). Such an increase in taste receptors is shared between *D. suzukii* and *D. biarmipes*, suggesting that an ancestral generalist feeding behavior characterized the whole suzukii subgroup, rather than *D. suzukii* alone.

The association between the burst in number of taste receptor genes and a change in ecology fits well with the observation that gene duplication is a major source of genetic, and phenotypic, novelty. After duplication, redundant genes may experience relaxed selection; their fate will then be defined by a combination of drift and selection, with retained duplications that will experience a distinct regime of purifying selection (Lynch and Conery 2000). Therefore, we can hypothesize that the proportion of duplicate genes in a genome is an excellent genetic proxy for adaptation to new habitats (Makino and Kawata 2012). The burst of GR and dIR duplications in the branch leading to the suzukii subgroup has a magnitude comparable only with the losses that occurred in GRs in the endemic and highly adapted *D. sechellia* lineage (McBride 2007; McBride *et al.* 2007); this suggests an adaptive role of GR and dIR duplications in expanding *D. suzukii* distribution to heterogeneous environments, likely promoting a generalist feeding behavior.

### Do selective constraints act on D. suzukii gustative receptors?

One interesting observation that emerges from our molecular evolution analysis is that, in *D. suzukii*, all chemoreceptor families (GRs, dIRs, and ORs) have a higher rate of nonsynonymous site evolution (*d_N_*) compared to that of 1021 nuclear genes estimated in a previous study (Ometto *et al.* 2013). In contrast, this was not observed for *D. biarmipes* chemoreceptor genes. Results obtained by Ometto *et al.* (2013) showed that *d_N_* and *d_S_* rates were both higher in *D. biarmipes* compared to *D. suzukii* and no differences in ω ratio existed between the two species. In our study, we observed an increased *d_S_* rate in *D. biarmipes* for all the gene families tested but, unexpectedly, no differences at *d_N_* rate distributions emerged between *D. biarmipes* and *D. suzukii*. This points toward an increased evolution rate acting on *D. suzukii* chemosensory proteins. The increased amino acid substitution rate might be explained by the presence of fewer selective constraints acting on *D. suzukii* chemoreceptors or by an increase in positive selection shaping the molecular evolution of specific GRs, dIRs, and ORs. In the second case, the fixation of specific genes would lead to a high dispersion in the distribution of *d_N_* across the gene family (Betancourt and Presgraves 2002). When we compared ω variances for each gene family between *D. suzukii* and *D. biarmipes*, we observed that such a hypothesis accounts only for GR*s* (Levene’s test: *F* = 5.40; df = 1131; and *P* = 0.022). In particular, gene targets of positive selection that are responsible for the higher variance are four duplicate *Ds*GRs (including the three GR59d8, see below) having a ω > 1 (Levene’s test without the four *Ds*GRs: *F* = 1.33; df = 1131; and *P* = 0.251). In cases of dIRs and ORs, whose variance do not differ between *D. suzukii* and *D. biarmipes*, the increased rate of amino acid substitutions may be due to pervasive relaxed selection, in accordance with what has been observed at the intra specific level in *D. melanogaster* (Arguello *et al.* 2016).

### Bitter taste receptors putatively relevant for the biology and control of D. suzukii

In terms of gene number, *D. suzukii* has only one more GR and one more dIR compared to *D. biarmipes*. However, differences are more pronounced because of species-specific isoforms characterizing some of the duplicate families. The most striking case is the extra duplications of GR59d8 which seems unique to *D. suzukii* (Figure 3); the exact affinity of these genes to their orthologs in *D. biarmipes* is hard to decipher and we cannot exclude an ancient origin on the common ancestor followed by different evolutionary histories in the two species. Regardless of their origin, these three genes are characterized by an ω > 1 only in *D. suzukii*, indicating strong positive selection acting on them (Table 1). We speculate that these paralogs may have played an active role in the adaption of *D. suzukii* to its fresh fruit polyphagous ovipositing behavior; we therefore consider them good candidates for functional studies and downstream practical application. Notably, these genes are expressed in heads and in the larval stage; therefore, we suggest that they may be involved in oviposition host choice and larval feeding behavior.

The limited knowledge (mostly restricted to *D. melanogaster*) about the function of most GRs and dIRs makes it difficult to propose a functional ecological role for duplications unique of *D. suzukii*, as well as for the other duplications shared with D. *biarmipes*. In fact, most genes whose function has been deorphanized in *D. melanogaster* do not exhibit copy number variation, with the notable exception of the L-canavanine receptor GR98b (Shim *et al.* 2015), which is duplicated only in *D. biarmipes*. However, most of the GRs duplicated in *D. suzukii* and/or in *D. biarmipes* (GR36b, GR59a, GR59d, GR93b, and GR98b) are expressed in bitter-sensing neurons in *D. melanogaster* (Weiss *et al.* 2011; Ling *et al.* 2014). This is concordant with what has been observed in *Heliconius* spp. and *D. sechellia*, where adaptive gene gain and loss appear to primarily affect GRs presumed to respond to bitter compounds (McBride 2007; McBride *et al.* 2007; Briscoe *et al.* 2013). Moreover, the proportion of putative bitter-related genes that experience a different selective pressure in both *D. suzukii* and *D. biarmipes* is particularly high (75 and 78% genes, respectively). In general, specialists tend to have a lower number of bitter receptors than generalists, since the latter enter into contact with a larger array of toxic molecules (McBride 2007; McBride *et al.* 2007; Briscoe *et al.* 2013). However, exceptions to this rule exist: *Bombyx mori*, which feeds exclusively on mulberry leaves, has an increased number of bitter-receptors probably used to detect the bitter compounds typical of its host plant (Wanner and Robertson 2008). We can hypothesize that the ancestor of the suzukii subgroup experienced a transition from one ecological niche to another that required the neo/subfunctionalization of newly duplicated genes to allow a wider recognition of bitter-related compounds.

Even more difficult is the prediction of the ecological function of duplicate dIRs, since less information is available compared to GRs. A family that is particularly enriched in *D. suzukii* (IR52), is mainly expressed in pheromone-sensing neurons located on forelegs in *D. melanogaster*, and *DmIR52c* is indeed required for normal copulation behavior (Koh *et al.* 2014). Duplication in *DsIR52c* in *D. suzukii* may be related to a different mating communication system specific for this species, since *D. suzukii* does not produce the sex pheromone *cis*-11-octadecenyl acetate (cVA), which is a pheromone basal to *Drosophila* species (Dekker *et al.* 2015). Expression studies also support the role of members of family IR52 as putative pheromone receptors in *D. suzukii*; the expression of all IR52 isoforms has been observed in thorax segments of *D. suzukii* adults, and more specifically in adult forelegs, but never in heads.

Expression of duplicate dIRs and GRs in different parts of the *D. suzukii* body (mainly in the head) is consistent with their role as taste receptors, since in the model *D. melanogaster* they are expressed in GRNs scattered along the body (Joseph and Carlson 2015). Within each family, each member was expressed with a different pattern, with the exception of the GR93d family, whose three members are expressed together in the three parts of the *D. suzukii* body. Considering the limitations of using RT-PCR to studying the expression of chemoreceptor genes (which only enabled us to examine tissue-specific and not neuron-specific patterns), our results suggest that duplicate GRs and dIRs in *D. suzukii* might have diverged their temporal and/or spatial expression after duplication, in response to neo-functionalization events.

### Conclusions

The analysis of gene gains/losses, molecular evolution, and expression patterns of *D. suzukii* tastant receptors has shown that GR and dIR gene families experience rapid gene family evolution. In particular, comparison with the closely related species *D. biarmipes* revealed a high number of gene gains occurred on the branch leading to the suzukii subgroup, whereas few specific genomic events (for instance the GR59d duplications) characterized the *D. suzukii* lineage. Overall, our results bring us one step closer to understanding *D. suzukii* innovative ecological behavior and provide a foundation for further studies aiming to disentangle the mechanisms of oviposition preferences, for example providing candidate taste receptors specific for *D. suzukii*, which may be tested for their ligand affinity and their role in the oviposition behavior of this species.

## Supplementary Material

Supplemental Material

## References

[bib1] Abascal F. Zardoya R. Telford M. J., 2010 TranslatorX: multiple alignment of nucleotide sequences guided by amino acid translations. Nucleic Acids Res. 38: 1–7. 20435676 10.1093/nar/gkq291 PMC2896173

[bib2] Almeida F. C. Sánchez-Gracia A. Campos J. L. Rozas J., 2014 Family size evolution in *Drosophila* chemosensory gene families: a comparative analysis with a critical appraisal of methods. Genome Biol. Evol. 6: 1669–1682. 24951565 10.1093/gbe/evu130 PMC4122928

[bib3] Arguello J. R. Cardoso-Moreira M. Grenier J. K. Gottipati S. Clark A. G., 2016 Extensive local adaptation within the chemosensory system following Drosophila melanogaster’s global expansion. Nat Commun. 7: ncomms11855. 27292132 10.1038/ncomms11855 PMC4910016

[bib4] Asplen M. K. Anfora G. Biondi A. Choi D. S. Chu D., 2015 Invasion biology of spotted wing Drosophila (*Drosophila suzukii*): a global perspective and future priorities. J. Pest Sci. 88: 469–494.

[bib5] Benjamini Y. Hochberg Y., 1995 Controlling the false discovery rate: a practical and powerful approach to multiple testing. J.R. Stat. Soc. 57: 289–300.

[bib6] Benton R. Vannice K. S. Gomez-Diaz C. Vosshall L. B., 2009 Variant ionotropic glutamate receptors as chemosensory receptors in *Drosophila*. Cell 136: 149–162. 19135896 10.1016/j.cell.2008.12.001 PMC2709536

[bib7] Betancourt A. J. Presgraves D. C., 2002 Linkage limits the power of natural selection in *Drosophila*. Proc. Natl. Acad. Sci. USA 99: 13616–13620. 12370444 10.1073/pnas.212277199 PMC129723

[bib8] Briscoe A. D. Macias-Muñoz A. Kozak K. M. Walters J. R. Yuan F., 2013 Female behaviour drives expression and evolution of gustatory receptors in butterflies. PLoS Genet. 9: e1003620. 23950722 10.1371/journal.pgen.1003620 PMC3732137

[bib9] Chiu J. C. Jiang X. Zhao L. Hamm C. A. Cridland J. M., 2013 Genome of *Drosophila suzukii*, the spotted wing drosophila. G3 (Bethesda) 3: 2257–2271. 24142924 10.1534/g3.113.008185 PMC3852387

[bib10] Clyne P. J. Warr C. G. Freeman M. R. Lessing D. Kim J., 1999 A novel family of divergent seven-transmembrane proteins: candidate odorant receptors in Drosophila. Neuron 22: 327–338. 10069338 10.1016/s0896-6273(00)81093-4

[bib11] Clyne P. J. Warr C. G. Carlson J. R., 2000 Candidate taste receptors in *Drosophila*. Science 287: 1830–1834. 10710312 10.1126/science.287.5459.1830

[bib12] Croset V. Rytz R. Cummins S. F. Budd A. Brawand D., 2010 Ancient protostome origin of chemosensory ionotropic glutamate receptors and the evolution of insect taste and olfaction. PLoS Genet. 6: e1001064. 20808886 10.1371/journal.pgen.1001064 PMC2924276

[bib13] Dekker T. Revadi S. Mansourian S. Ramasamy S. Lebreton S., 2015 Loss of *Drosophila* pheromone reverses its role in sexual communication in *Drosophila suzukii*. Proc. Biol. Sci. 282: 20143018. 25716789 10.1098/rspb.2014.3018 PMC4375874

[bib14] Edgar R. C., 2004 MUSCLE: multiple sequence alignment with high accuracy and high throughput. Nucleic Acids Res. 32: 1792–1797. 15034147 10.1093/nar/gkh340 PMC390337

[bib15] Erdelyan C. N. G. Mahood T. H. Bader T. S. Y. Whyard S., 2012 Functional validation of the carbon dioxide receptor genes in *Aedes aegypti* mosquitoes using RNA interference. Insect Mol. Biol. 21: 119–127. 22122783 10.1111/j.1365-2583.2011.01120.x

[bib16] Freeman E. G. Dahanukar A., 2015 Molecular neurobiology of *Drosophila* taste. Curr. Opin. Neurobiol. 34: 140–148. 26102453 10.1016/j.conb.2015.06.001 PMC4577450

[bib17] French A. Moutaz A. A. Mitra A. Yanagawa A. Sellier M.-J., 2015 *Drosophila* bitter taste(s). Front. Integr. Nuerosci. 9: 1–13. 10.3389/fnint.2015.00058 PMC4658422 26635553

[bib18] Gao Q. Chess A., 1999 Identification of candidate Drosophila olfactory receptors from genomic DNA sequence. Genomics 60: 31–39. 10458908 10.1006/geno.1999.5894

[bib19] Gardiner A. Barker D. Butlin R. K. Jordan W. C. Ritchie M. G., 2008 *Drosophila* chemoreceptor gene evolution: selection, specialization and genome size. Mol. Ecol. 17: 1648–1657. 18371013 10.1111/j.1365-294X.2008.03713.x

[bib20] Guo S. Kim J., 2007 Molecular evolution of *Drosophila* odorant receptor genes. Mol. Biol. Evol. 24: 1198–1207. 17331958 10.1093/molbev/msm038

[bib21] Hall T., 1999 BioEdit: a user-friendly biological sequence alignment editor and analysis program for Windows 95/98/NT. Nucleic Acids Symp. Ser. 41: 95–98.

[bib22] Jones W. D. Cayirlioglu P. Kadow I. G. Vosshall L. B., 2007 Two chemosensory receptors together mediate carbon dioxide detection in *Drosophila*. Nature 445: 86–90. 17167414 10.1038/nature05466

[bib23] Joseph R. M. Carlson J. R., 2015 *Drosophila* chemoreceptors: a molecular interface between the chemical world and the brain. Trends Genet. 31: 683–695. 26477743 10.1016/j.tig.2015.09.005 PMC4674303

[bib24] Koh T. W. He Z. Gorur-Shandilya S. Menuz K. Larter N. K., 2014 The *Drosophila* IR20a clade of ionotropic receptors are candidate taste and pheromone receptors. Neuron 83: 850–865. 25123314 10.1016/j.neuron.2014.07.012 PMC4141888

[bib25] Kondrashov F. A Rogozin I. B. Wolf Y. I. Koonin E. V, 2002 Selection in the evolution of gene duplications. Genome Biol. 3: RESEARCH0008. 11864370 10.1186/gb-2002-3-2-research0008 PMC65685

[bib26] Kwon J. Y. Dahanukar A. Weiss L. A. Carlson J. R., 2007 The molecular basis of CO_2_ reception in *Drosophila*. Proc. Natl. Acad. Sci. USA 104: 3574–3578. 17360684 10.1073/pnas.0700079104 PMC1805529

[bib27] Kwon J. Y. Dahanukar A. Weiss L. A. Carlson J. R., 2011 Molecular and cellular organization of the taste system in the *Drosophila* larva. J. Neurosci. 31: 15300–15309. 22031876 10.1523/JNEUROSCI.3363-11.2011 PMC3225198

[bib54] Lee J. C. Bruck D. J. Curry H. Edwards D. Haviland D. R., 2011 The susceptibility of small fruits and cherries to the spotted-wing drosophila, *Drosophila suzukii*. Pest Manag. Sci. 67: 1358–1367. 21710685 10.1002/ps.2225

[bib28] Letunic I. Bork P., 2007 Interactive Tree Of Life (iTOL): an online tool for phylogenetic tree display and annotation. Bioinformatics 23: 127–128. 17050570 10.1093/bioinformatics/btl529

[bib29] Librado P. Vieira F. G. Rozas J., 2012 BadiRate: estimating family turnover rates by likelihood-based methods. Bioinformatics 28: 279–281. 22080468 10.1093/bioinformatics/btr623

[bib30] Ling F. Dahanukar A. Weiss L. A. Kwon J. Y. Carlson J. R., 2014 The molecular and cellular basis of taste coding in the legs of *Drosophila*. J. Neurosci. 34: 7148–7164. 24849350 10.1523/JNEUROSCI.0649-14.2014 PMC4028494

[bib31] Löytynoja A. Goldman N., 2005 An algorithm for progressive multiple alignment of sequences with insertions. Proc. Natl. Acad. Sci. USA 102: 10557–10562. 16000407 10.1073/pnas.0409137102 PMC1180752

[bib32] Lynch M. Conery J. S., 2000 The evolutionary fate and consequences of duplicate genes. Science 290: 1151–1155. 11073452 10.1126/science.290.5494.1151

[bib33] Makino T. Kawata M., 2012 Habitat variability correlates with duplicate content of *Drosophila* genomes. Mol. Biol. Evol. 29: 3169–3179. 22586328 10.1093/molbev/mss133 PMC3457775

[bib34] McBride C. S., 2007 Rapid evolution of smell and taste receptor genes during host specialization in *Drosophila sechellia*. Proc. Natl. Acad. Sci. USA 104: 4996–5001. 17360391 10.1073/pnas.0608424104 PMC1829253

[bib35] McBride C. S. Arguello J. R. O’ Meara B. C., 2007 Five *Drosophila* genomes reveal nonneutral evolution and the signature of host specialization in the chemoreceptor superfamily. Genetics 177: 1395–1416. 18039874 10.1534/genetics.107.078683 PMC2147975

[bib36] Missbach C. Dweck H. K. M. Vogel H. Vilcinskas A. Stensmyr M. C., 2014 Evolution of insect olfactory receptors. eLife 3: e02115. 24670956 10.7554/eLife.02115 PMC3966513

[bib37] Ni L. Bronk P. Chang E. C. Lowell A. M. Flam J. O., 2013 A gustatory receptor paralogue controls rapid warmth avoidance in *Drosophila*. Nature 500: 580–584. 23925112 10.1038/nature12390 PMC3758369

[bib38] Ometto L. Cestaro A. Ramasamy S. Grassi A. Revadi S., 2013 Linking genomics and ecology to investigate the complex evolution of an invasive *Drosophila* pest. Genome Biol. Evol. 5: 745–757. 23501831 10.1093/gbe/evt034 PMC3641628

[bib39] Poyet M. Le Roux V. Gibert P. Meirland A. Prévost G., 2015 The wide potential trophic niche of the asiatic fruit fly *Drosophila suzukii*: the key of its invasion success in temperate Europe? PLoS One 10: e0142785. 26581101 10.1371/journal.pone.0142785 PMC4651357

[bib40] Ramasamy S. Ometto L. Crava C. M. Revadi S. Kaur R., 2016 The evolution of olfactory gene families in *Drosophila* and the genomic basis of chemical-ecological adaptation in *Drosophila suzukii*. Genome Biol. Evol. 8: 2297–2311. 27435796 10.1093/gbe/evw160 PMC5010897

[bib41] Robertson H. M., 2015 The insect chemoreceptor superfamily is ancient in animals. Chem. Senses 40: 609–614. 26354932 10.1093/chemse/bjv046

[bib42] Robertson H. M. Warr C. G. Carlson J. R., 2003 Molecular evolution of the insect chemoreceptor gene superfamily in *Drosophila melanogaster*. Proc. Natl. Acad. Sci. USA 100: 14537–14542. 14608037 10.1073/pnas.2335847100 PMC304115

[bib43] Rota-Stabelli O. Blaxter M. Anfora G., 2013 Drosophila suzukii. Curr. Biol. 23: R8–R9. 23305672 10.1016/j.cub.2012.11.021

[bib44] Sato K. Tanaka K. Touhara K., 2011 Sugar-regulated cation channel formed by an insect gustatory receptor. Proc. Natl. Acad. Sci. USA 108: 11680–11685. 21709218 10.1073/pnas.1019622108 PMC3136286

[bib45] Shim J. Lee Y. Jeong Y. T. Kim Y. Lee M. G., 2015 The full repertoire of *Drosophila* gustatory receptors for detecting an aversive compound. Nat. Commun. 6: 8867. 26568264 10.1038/ncomms9867 PMC4660205

[bib46] Stamatakis A., 2014 RAxML version 8 a tool for phylogenetic analysis and post-analysis of large phylogenies. Bioinformatics 30: 1312–1313. 24451623 10.1093/bioinformatics/btu033 PMC3998144

[bib47] Stewart S. Koh T. Ghosh A. C. Carlson J. R., 2015 Candidate ionotropic taste receptors in the *Drosophila* larva. Proc. Natl. Acad. Sci. USA 112: 4195–4201. 25825777 10.1073/pnas.1503292112 PMC4394268

[bib48] Stocker R. F., 1994 The organization of the chemosensory system in *Drosophila melanogaster*: a review. Cell Tissue Res. 275: 3–26. 8118845 10.1007/BF00305372

[bib49] Vosshall L. B. Amrein H. Morozov P. S. Rzhetsky A. Axel R., 1999 A spatial map of olfactory receptor expression in the Drosophila antenna. Cell 96: 725–736. 10089887 10.1016/s0092-8674(00)80582-6

[bib50] Wanner K. W. Robertson H. M., 2008 The gustatory receptor family in the silkworm moth *Bombyx mori* is characterized by a large expansion of a single lineage of putative bitter receptors. Insect Mol. Biol. 17: 621–629. 19133074 10.1111/j.1365-2583.2008.00836.x

[bib51] Weiss L. A. Dahanukar A. Kwon J. Y. Banerjee D. Carlson J. R., 2011 The molecular and cellular basis of bitter taste in *Drosophila*. Neuron 69: 258–272. 21262465 10.1016/j.neuron.2011.01.001 PMC3033050

[bib52] Yang Z., 2007 PAML 4: phylogenetic analysis by maximum likelihood. Mol. Biol. Evol. 24: 1586–1591. 17483113 10.1093/molbev/msm088

[bib53] Yu D. Zalom F. G. Hamby K. A., 2013 Host status and fruit odor response of *Drosophila suzukii* (Diptera: Drosophilidae) to figs and mulberries. J. Econ. Entomol. 106: 1932–1937. 24020313 10.1603/ec12480

