# Supplementary material for: Evolutionary Insights into Taste Perception of the Invasive Pest Drosophila suzukii
Source: G3 (Bethesda). 2016 Oct 19;6(12):4185–96. doi: 10.1534/g3.116.036467 (PMC5144986; doi:10.1534/g3.116.036467)
Supplement: Supplemental Material [file supp_6_12_4185__index.html]

Evolutionary Insights into Taste Perception of the Invasive Pest Drosophila suzukii — Supplemental Material 

# Evolutionary Insights into Taste Perception of the Invasive Pest *Drosophila suzukii*

## Supplemental Material for Crava *et al.*, 2016

**Files in this Data Supplement:**

- File S5 - PDF file containing supplementary figures S1, S2 and S3. (.pdf, 408 KB)
- File S1 - *D. suzukii* GR and dIR predicted transcripts. Sequences are in FASTA format. (.zip, 52 KB)
- File S2 - *D. biarmipes* GR and dIR predicted transcripts. Sequences are in FASTA format. (p) after the gene name indicates a sequence considered a pseudogene due to an early stop codon present in the ORF. (.zip, 53 KB)
- File S3 - Multiple sequence alignment used for phylogeny shown in Figure 1. The alignment after cleaning is shown in FASTA format. (.zip, 54 KB)
- File S4 - Multiple sequence alignment used for phylogeny shown in Figure 2. The alignment after cleaning is shown in FASTA format. (.zip, 58 KB)
- Table S1 - List of GRs and dIRs identified in *D. biarmipes* and *D. suzukii* genomes. (.xls, 62 KB)
- Table S2 - Nomenclature of GR and IR genes used in phylogenetic and molecular evolution analysis. (.xls, 68 KB)
- Table S3 - Gene matrix used for birth-death analysis. (.xls, 36 KB)
- Table S4 - Sequences of primers used in RT-PCR. (.xlsx, 12 KB)
- Table S5 - List of genes identified after molecular evolution analysis. (.xls, 41 KB)
