# Supplementary material for: Evolutionary Insights into Taste Perception of the Invasive Pest Drosophila suzukii
Source: G3 (Bethesda). 2016 Oct 19;6(12):4185–96. doi: 10.1534/g3.116.036467 (PMC5144986; doi:10.1534/g3.116.036467)
Supplement: Supplemental Material [file supp_g3.116.036467_FileS5.pdf]

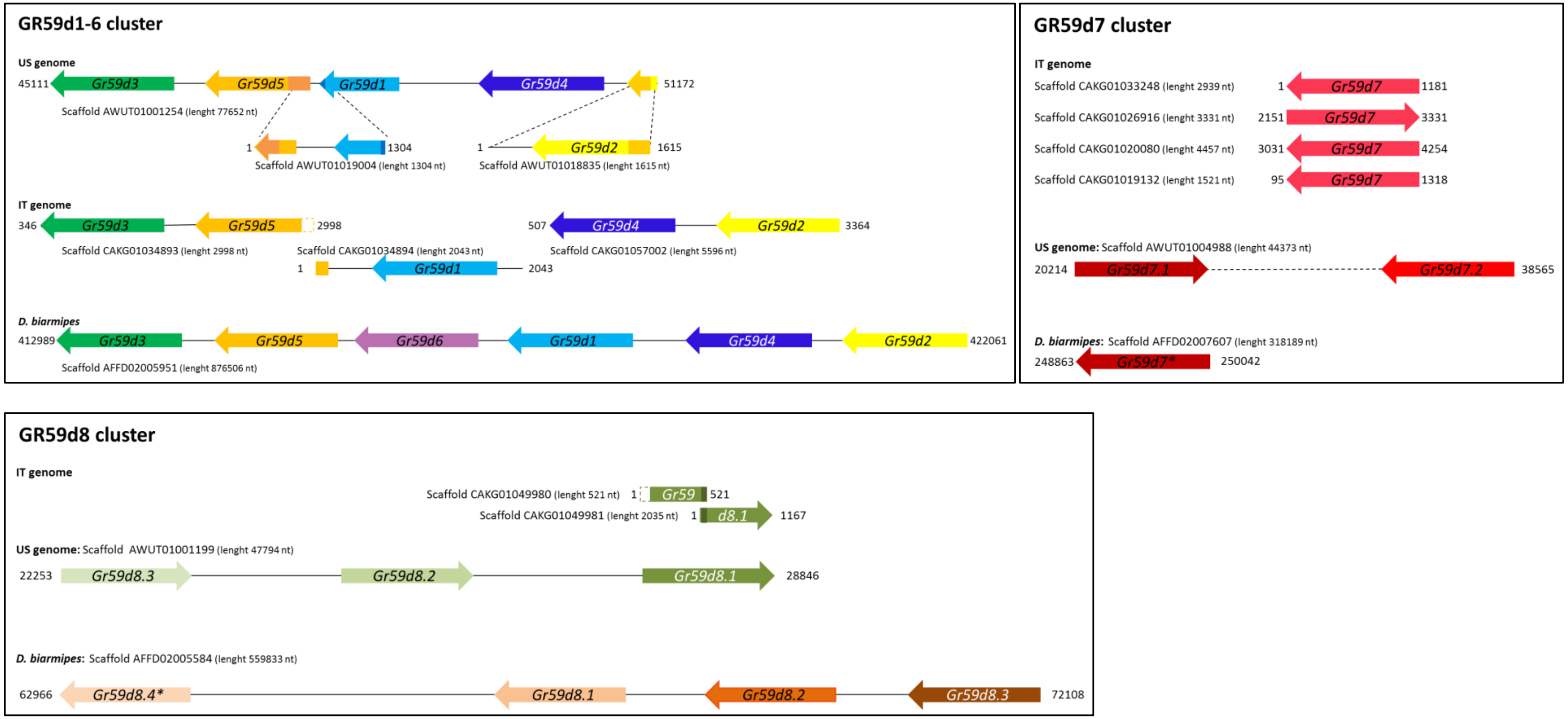

Figure S1. Synteny conservation in GR59d gene family.

The figure shows gene organization of the GR59d family in *D. suzukii* and *D. biarmipes*, with homologous genes drawn in the same color. Black continuous lines indicate intergenic regions as well as black dotted lines; in this last case they are not proportional to scaffold length. Overlapping gene regions shared between two scaffolds are highlighted with darker colors. Dotted boxes indicate missing gene region in that scaffold. Gene names followed by an asterisk indicate pseudogenes. Scaffold names refer to Genbank accession numbers. Duplicated *DsGR59d7* and *DsGR59d8* were retrieved only in the American genome (US) and not in the most fragmented Italian one (IT)

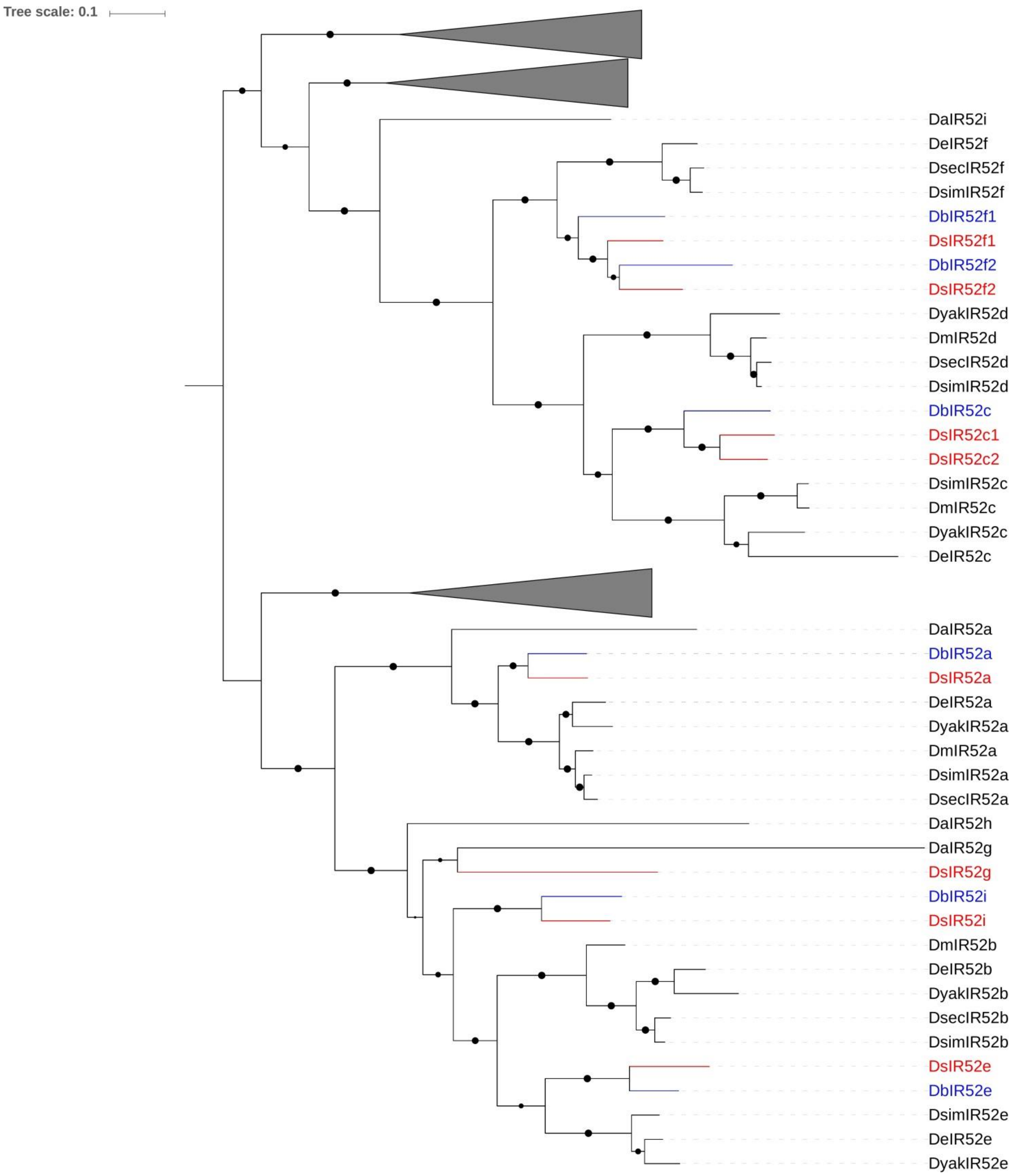

Figure S2. Gene phylogeny of IR52 family from fourteen Drosophila species.

Maximum likelihood tree calculated with RAxML and based on Muscle nucleotide alignment implemented in TranslatorX. Phylogenetic relationships of all genes included in gene family IR52 and used in birth death-analysis are displayed in the tree. Bootstrap support is out of 100 replicates and support >80 is indicated by black dots whose sizes are according to bootstrap values. Branches highlighted in red show *D. suzukii* genes and branches highlighted in blue, *D. biarmipes* genes. Branches without *D. suzukii* or *D. biarmipes* genes were collapsed.

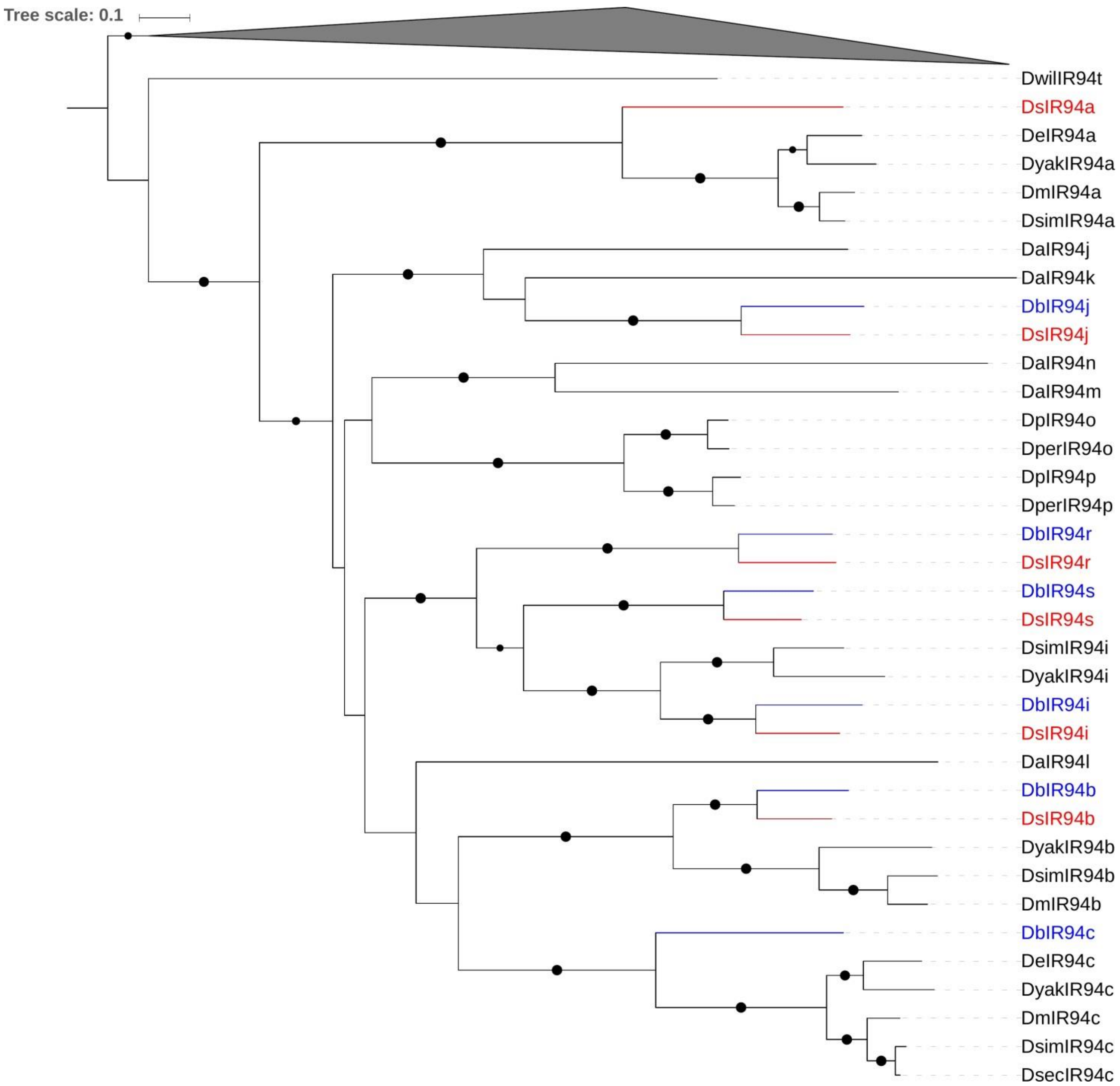

Figure S3. Gene phylogeny of IR94 family from fourteen Drosophila species.

Maximum likelihood tree calculated with RAxML and based on Muscle nucleotide alignment implemented in TranslatorX. Phylogenetic relationships of all genes included in gene family IR94 and used in birth death-analysis are displayed in the tree. Bootstrap support is out of 100 replicates and support >80 is indicated by black dots whose sizes are according to bootstrap values. Branches highlighted in red show *D. suzukii* genes and branches highlighted in blue, *D. biarmipes* genes. Branches without *D. suzukii* or *D. biarmipes* genes were collapsed.
